# Selection analyses of paired HIV-1 *gag* and *gp41* sequences obtained before and after antiretroviral therapy

**DOI:** 10.1038/sdata.2018.147

**Published:** 2018-07-24

**Authors:** Philip L. Tzou, Soo-Yon Rhee, Sergei L. Kosakovsky Pond, Justen Manasa, Robert W. Shafer

**Affiliations:** 1 Division of Infectious Diseases, Department of Medicine Stanford University, Stanford, CA 94305, USA; 2 Department of Biology, Temple University, Philadelphia, PA 19122, USA

**Keywords:** HIV infections, Quality control, Viral evolution, Data processing, RNA sequencing

## Abstract

Most HIV-1-infected individuals with virological failure on a pharmacologically-boosted protease inhibitor (PI) regimen do not develop PI-resistance protease mutations. One proposed explanation is that HIV-1 *gag* or *gp41* cytoplasmic domain mutations might also reduce PI susceptibility. In a recent study of paired *gag* and *gp41* sequences from individuals with virological failure on a PI regimen, we did not identify PI-selected mutations and concluded that if such mutations existed, larger numbers of paired sequences from multiple studies would be needed for their identification. In this study, we generated site-specific amino acid profiles using *gag* and *gp41* published sequences from 5,338 and 4,242 ART-naïve individuals, respectively, to assist researchers identify unusual mutations arising during therapy and to provide scripts for performing established and novel maximal likelihood estimates of dN/dS substitution rates in paired sequences. The pipelines used to generate the curated sequences, amino acid profiles, and dN/dS analyses will facilitate the application of consistent methods to paired *gag* and *gp41* sequence datasets and expedite the identification of potential sites under PI-selection pressure.

## Background & Summary

HIV-1 protease mutations responsible for protease inhibitor (PI) resistance are now uncommon in patients with virological failure on an initial PI-containing regimen, particularly regimens including pharmacologically-boosted lopinavir, atazanavir, or darunavir^1–3^ One explanation for the infrequent occurrence of PI-resistance mutations in protease is that mutations outside of protease might reduce PI susceptibility even in the absence of primary PI resistance protease mutations. Indeed, many studies have reported that *gag* cleavage and non-cleavage site mutations may compensate for the reduced fitness associated with primary PI-resistance protease mutations^4–11^ and several studies of pseudotyped viruses reported that genetic loci in matrix (MA) *gag*^12,13^ and in the *gp41* cytoplasmic domain (CD)^14^ can reduce PI susceptibility in the absence of PI-resistance protease mutations.

To identify *gag* and *gp41* mutations under selective PI pressure, we recently sequenced *gag* and/or *gp41* in 61 individuals with virological failure on a PI or a control nonnucleoside RT inhibitor (NNRTI) containing regimen^15^. We quantified nonsynonymous and synonymous mutations in both genes and identified sites exhibiting signal for directional or diversifying selection. We also used published *gag* and *gp41* polymorphism data to highlight mutations displaying a high selection index, defined as changing from a conserved or common amino acid variant to an uncommon amino acid variant. The rationale for this latter analysis is that most drug-resistance mutations in established targets of antiviral therapy including protease, RT, integrase, and the extracellular domain of *gp41* are amino acid variants at sites that are non-polymorphic in the absence of selective drug pressure.

In our previous study, many amino acid mutations were found to emerge in *gag* and in *gp41*-CD in both the PI- and NNRTI-treated groups. However, in neither gene, were there discernible differences between the two groups in overall numbers of mutations, mutations displaying evidence of diversifying or directional selection, or mutations with a high selection index. Based on this previous study, we concluded that if *gag* and/or *gp41* encoded PI-resistance mutations, they might not be confined to repeated mutations at a few sites, and that multiple studies with large numbers of paired sequences from individuals with virological failure on a PI-containing regimen would need to be pooled to identify such mutations. To facilitate such studies, we provide here a detailed description of the methods used to generate the datasets and analytic results used in our previous study.

The selection index analyses, in particular, require additional exposition because they used data derived from the curation and annotation of *gag* and *gp41* sequences from more than 500 GenBank submission sets and/or peer-reviewed publications to determine the polymorphism rates at each *gag* and *gp41* position. The annotation of these references according to the treatment status of the individuals in the references is included as part of this manuscript’s Data Citation. The selection index analyses, also required performing quality control analyses of each *gag* and *gp41* sequence and determining the prevalence of each mutation at each position. Finally, the HyPhy scripts described in this manuscript make it possible to exactly replicate each of the maximum likelihood estimates of the ratio of non-synonymous and synonymous substitution rates presented in our original manuscript.

## Methods

### *Gag* and *gp41* sequences of paired samples obtained before and after PI or NNRTI therapy

The sequences described in our previous manuscript were obtained from HIV-1-infected individuals in Northern California who had genotypic resistance tests performed between April 2001 and June 2013 and from participants in the ACTG A5202 clinical trial^3,15^. The sequences were derived from plasma virus samples obtained before and after therapy from 41 previously PI-naïve subjects who had received a ritonavir-boosted PI-containing regimen or from 20 control subjects who received an NNRTI-containing regimen. Among the 41 PI-treated subjects, paired sequences before and after PI treatment were available for both *gag* and *gp41* in 11 individuals, for *gag* alone in 13 individuals, and for *gp41* alone in 17 individuals. Among 20 NNRTI control subjects, paired sequences before and after NNRTI treatment were available for both *gag* and *gp41* in 13 individuals, for *gag* alone in three individuals, and *gp41* alone in four individuals. Table 1 (available online only) contains the GenBank accessions for each of the paired protease, *gag* and *gp41* sequences from the 41 PI- and 20 NNRTI-treated individuals. This study was approved by the Institutional Review Boards (IRBs) of Stanford University, KPNC, and the NIH ACTG and all study methods were performed in accordance with the guidelines of these IRBs. Informed consent was required for participation in the ACTG 5202 trial. The Stanford University and KPNC IRBs provided a waiver of informed consent for the study of remnant KPNC samples that were unlinked to individual protected health information.

The plasma samples were processed and underwent direct PCR Sanger sequencing as described in our previous manuscript^15^. Each *gag*, and *gp41* sequence was aligned using the Translation Align option with the ClustalW algorithm for multiple sequence alignment using the Geneious R11 software^16^. The parameters used were the default values (cost matrix: BLOSUM; gap open cost: 10; gap extend cost: 0.1). The multiple sequence alignment was then manually edited using the subtype B consensus sequence^17^. Manual edits were required for the *gag* but not the *gp41* sequence because *gag* contained many more indels than *gp41*. The manual edits were primarily the shifting of indels to be consistent with the remaining sequences and the subtype B consensus. Nucleotide insertions were then stripped from the sequence prior to subsequent analyses.

The original and aligned sets of FASTA sequences for *gag* and *gp41* are in the files gagOriginal.fas, gagAligned.fas, gp41Original.fas, and gp41Aligned.fas (Data Citation 1). The insertions in *gag* and *gp41* are in file insertions.csv. Neighbor-joining trees for each gene, created by HyPhy version 2.3.2 using the TN93 distance, confirmed that each pair of sequences clustered by individual. All trees (in Newick format) are included in Data Citation 1. Data Citation 1 also contains the initial and edited *gag* gene alignments in data/gag.geneious (Data Citation 1).

### Pairwise sequence comparisons and dN/dS analyses of *gag* and *gp41*

Tables 2 and 3 summarize the median proportions of pairwise nucleotide and amino acid changes, pairwise dN/dS ratios, and median proportions of IUPAC ambiguities in the pre- and post-treatment *gag* and *gp41* sequences, respectively. Pairwise dN/dS ratio estimation was implemented using a custom HyPhy v2.3.2 script scripts/pairwise-estimator-dnds.bf (Data Citation 1); this script reads in a collection of aligned coding sequences, splits them into host pairs (based on patient ID encoded in the FASTA sequence name), and estimates dN/dS by maximum likelihood using the MG94xREV codon substitution model^18^. The script also optionally restricts the analysis to a contiguous region of the alignment, for example to focus on a specific protein domain.

As noted in the previous manuscript, there was no difference in median proportions of pairwise nucleotide and amino acid changes and pairwise dN/dS ratios in *gag* and *gp41* between the PI- and NNRTI-treated patients. Also, as previously noted, there was a significant reduction in the median proportion of ambiguous nucleotides in *gag* between baseline and follow-up among the PI-treated patients: 0.4% (IQR:0.1% to 0.8%) vs 0.0% (IQR:0% to 0.6%; p=0.02 Mann-Whitney U test). Table 4 lists each of the amino acid changes that occurred at *gag* cleavage sites.

### Positional dN/dS selection analyses of *gag* and *gp41*

We ran the fixed effects likelihood (FEL) method, as implemented in HyPhy v2.3.2, to detect codon sites exhibiting diversifying selection in *gag* and *gp41* on the post-treatment branches using a p-value of 0.05 (refs 18,19). This analysis requires annotated phylogenetic trees (e.g., internalFiles/phylo/gagNNRTIs.tre (Data Citation 1)), i.e. trees where post-treatment branches are marked for testing. A convenient tool for annotating trees can be found at http://phylotree.hyphy.org. We also used Hyphy v2.3.2 to fit a model of episodic directional selection (MEDS) to the post-treatment branches pressure, also using a p-value of 0.05 (ref. 20). Table 5 shows the *gag* positions with evidence of diversifying selection and the *gag* mutations with evidence of directional selection within the PI and NNRTI-treatment groups. Table 6 shows the *gp41* positions with evidence of diversifying selection and evidence of directional selection within the PI and NNRTI-treatment groups. Both of these analyses identify candidate positions and mutations that are most likely to be under selective drug pressure. However, as is the case with any statistical testing procedures, it is possible that some of the positions and/or mutations are misclassified as either false positives or false negatives. Files containing shell scripts are provided to enable users to repeat these selection analyses with the same set of parameters that we used.

### Collection of previously published *gag* sequences from ARV-naïve individuals

**Figure 1:**
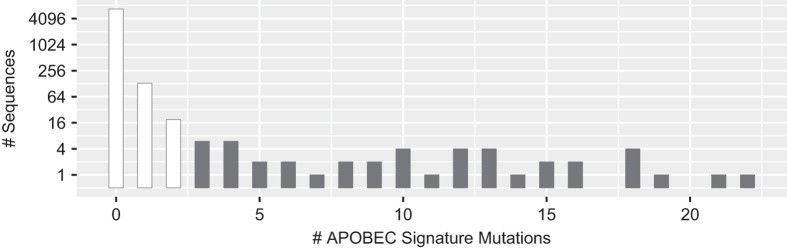
Distribution of *gag* APOBEC signature mutations. Distribution of the number of APOBEC signature *gag* mutations in the complete set of 7,031 one-per-person, quality-control filtered (i.e. sequences with large numbers of missing nucleotides and frame shifts were excluded) aligned complete *gag* sequences downloaded from the LANL HIV Sequence Database. The 46 sequences containing ≥3 APOBEC signature mutations were considered to be at high risk of having been subject to APOBEC-mediated G-to-A hypermutation and were excluded from our amino acid prevalence calculations.

We downloaded the complete set of 7,550 one-per-person aligned complete *gag* sequences from the Los Alamos National Laboratories (LANL) HIV Sequence Database^21^. We filtered 565 sequences that contained either large deletions or missing nucleotides (n=238), more than 3 frameshift mutations (n=281), or 3 or more signature APOBEC mutations (n=46) defined as mutations at highly conserved positions that were likely to occur in sequences containing stop codons and that occurred in an appropriate dinucleotide context: GG→AG for APOBEC3G GA→AA for APOBEC3F (ref. 22). Applying the Local FDR Poisson distribution using the R LocFDRPois package to our data, we found that presence of ≥3 signature APOBEC mutations was associated with a 0.99 likelihood of a sequence having undergone APOBEC-mediated G to A hypermutation^23^. Table 7 lists the 45 signature APOBEC mutations that we identified and Fig. 1 shows the distribution of the number of signature APOBEC mutations per *gag* sequence.

We then used Batch Entrez to submit each of the Accession IDs to GenBank^24^ and parsed the XML results to aggregate sequences into GenBank submission sets, henceforth referred to as studies, sharing either the same PubMed ID or the same Title and Author List fields. We reviewed the 264 studies reporting three or more individuals. Of these studies, 164 (62.1%) comprised solely ART-naïve individuals, 75 (28.4%) comprised individuals whose treatment status was unknown, and 25 (9.5%) comprised individuals who were ART-experienced. A summary of these 264 studies is provided in gagStudies.csv (Data Citation 1).

**Figure 2:**
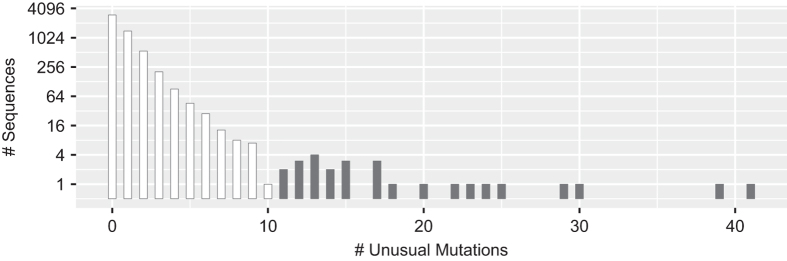
Distribution of unusual *gag* mutations. Distribution of the number of unusual *gag* amino acids in the 5,365 one-per-person, quality-control and APOBEC-filtered aligned complete *gag* sequences from PI-naïve individuals. Unusual amino acids were defined as those occurring in ≤0.1% of sequences. The 27 sequences containing ≥11 unusual amino acids were removed from the final PI-naïve *gag* amino acid profile.

**Figure 3:**
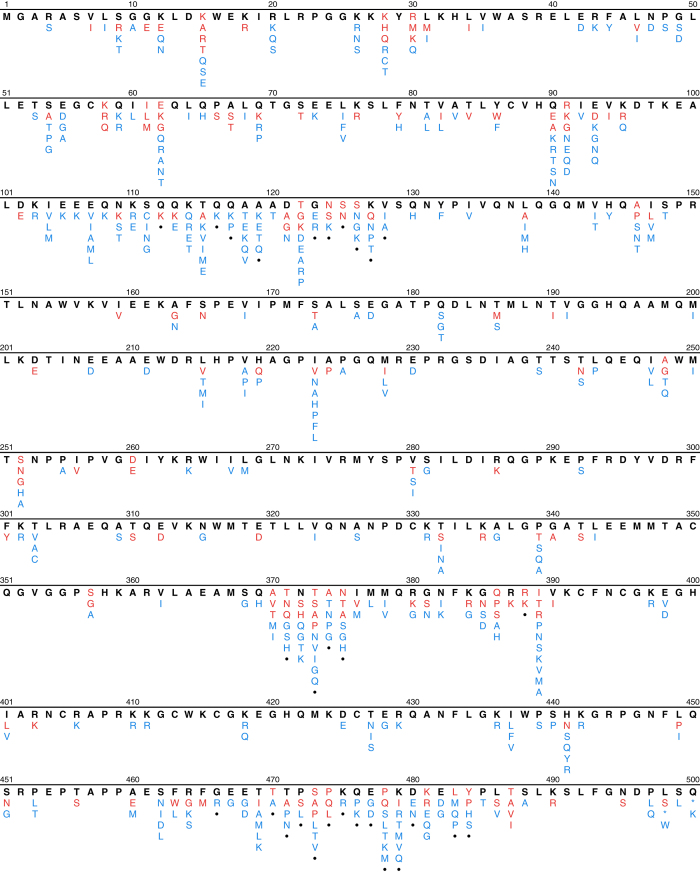
Distribution of HIV-1 group M *gag* amino acid variants in sequences from PI-naïve individuals. Amino acid variants occurring in 5,338 one-per-person sequences from PI-naïve individuals. Amino acids occurring in ≥50% of sequences are shown in bold black; those occurring in 10 to 49% of sequences are shown in red; and those occurring in 1 to 9% of sequences are shown in blue. Positions at which insertions or deletions have been reported in ≥1% of sequences are indicated by dots. The complete summary of all amino acid variants in group M sequences and for the most common subtypes can be found in the file naiveStudies/gagAAPrevalence.csv (Data Citation 1).

We then determined the proportion of each amino acid at each position in *gag* for the complete set of 5,365 one-per-person group M ART-naïve sequences as well as for the four LANL-designated subtypes (A, B, C, and CRF01_AE) for which at least 100 sequences were present. Site-specific amino acids present in 0.1% or fewer sequences were considered unusual. Fig. 2 shows that the numbers of *gag* sequences according to the number of unusual mutations per sequence monotonically decreases until n=10 unusual mutations. Therefore, we excluded sequences containing ≥11 unusual mutations since these 27 sequences were considered to be at high risk of poor sequence quality. We then recalculated the proportion of each amino acid at each position for the remaining 5,338 sequences. The original and aligned sets of FASTA sequences for the 5,338 one-per-person filtered sequences from these studies are in gagNaiveOriginal.fas and gagNaiveAligned.fas (Data Citation 1). The header for each sequence contains the GenBank accession number and the LANL-designated subtype. The file gagAAPrevalence.csv (Data Citation 1) lists the proportion of each amino acid at each *gag* position. In this file insertions, deletions, and mixtures are indicated by “ins”, “del”, and “X”, respectively. Figure 3 displays the distribution of amino acids at each of the 500 *gag* positions in the one-per-person group M HIV-1 *gag* sequences from ARV-naïve individuals.

### Collection of previously published *gp41* sequences from ARV-naïve individuals

**Figure 4:**
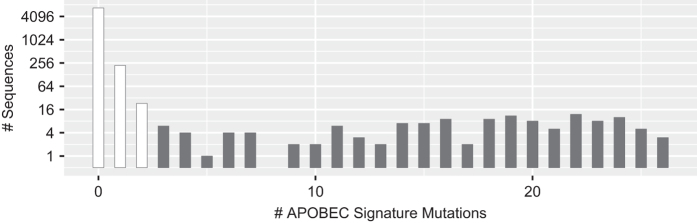
Distribution of *gp41* APOBEC signature mutations. Distribution of the number of *gp41* APOBEC signature mutations in the complete set of 7,166 one-per-person quality-control filtered (i.e. sequences with large numbers of missing nucleotides and frame shifts were excluded) aligned complete *gp41* sequences from the LANL HIV sequence database. The 130 sequences containing ≥3 APOBEC signature mutations were considered to be at high risk of having been subject to APOBEC-mediated G-to-A hypermutation and were excluded from our amino acid prevalence calculations.

We downloaded the complete set of 7,489 one-per-person aligned complete *gp41* sequences from the LANL HIV Sequence Database^21^. We filtered 453 sequences that contained either large deletions or missing nucleotides (n=234), more than 3 frameshift mutations (n=89), or 3 or more signature APOBEC mutations (n=130) defined as mutations at highly conserved positions that were likely to occur in sequences containing stop codons and that occurred in an appropriate dinucleotide context^22^. Applying the Local FDR Poisson distribution using the R LocFDRPois package to our data, we found that presence of ≥3 signature APOBEC mutations was associated with 0.97 likelihood of a sequence having undergone APOBEC-mediated G to A hypermutation^23^. Table 8 lists the 47 signature APOBEC mutations and Fig. 4 shows the distribution of the number of signature APOBEC mutations per sequence.

We then used Batch Entrez to submit each of the Accession IDs to GenBank^24^ and parsed the XML results to aggregate sequences into GenBank submission sets (or studies) sharing either the same PubMed ID or the same Title and Author List fields. We reviewed the 329 studies reporting five or more individuals. Of these studies, 191 (58.1%) comprised solely ART-naïve individuals, 95 (28.9%) comprised individuals whose treatment status was unknown, and 43 (13.0%) comprised individuals who were ART-experienced. A summary of these 329 studies is provided in gp41Studies.csv (Data Citation 1).

**Figure 5:**
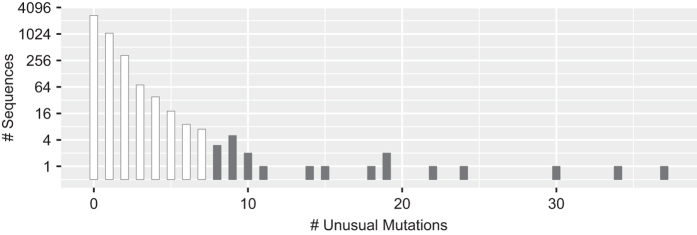
Distribution of unusual *gp41* mutations. Distribution of the number of unusual *gp41* amino acids in the 4,263 one-per-person quality-control and APOBEC-filtered aligned complete *gp41* sequences from PI-naïve individuals. Unusual amino acids were defined as those occurring in ≤0.1% of sequences. The 21 sequences containing ≥8 unusual amino acids were removed from the final PI-naïve *gp41* amino acid profile.

**Figure 6:**
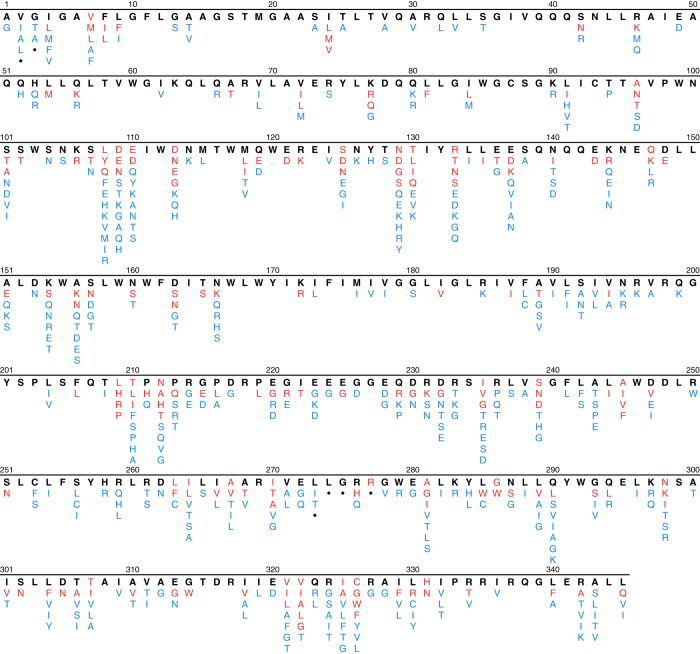
Distribution of HIV-1 group M *gp41* amino acid variants in sequences from PI-naïve individuals. Amino acid variants occurring in 4,242 one-per-person sequences from PI-naïve individuals. Amino acids occurring in ≥50% of sequences are shown in bold black; those occurring in 10 to 49% of sequences are shown in red; and those occurring in 1 to 9% of sequences are shown in blue. Positions at which insertions or deletions have been reported in ≥1% of sequences are indicated by dots. The complete summary of all amino acid variants in group M sequences and for the most common subtypes can be found in the file naiveStudies/gp41AAPrevalence.csv (Data Citation 1).

We then determined the proportion of each amino acid at each position in *gp41* for the complete set of 4,263 one-per-person group M ART-naïve sequences as well as for each of the four LANL-designated subtypes (A, B, C, and CRF01_AE) for which at least 100 sequences were present. Amino acids occurring at a proportion ≤0.1% were considered unusual. Figure 5 shows that the numbers of *gp41* sequences according to the number of unusual mutations per sequence monotonically decreases until n=7 unusual mutations. Therefore, we excluded sequences containing ≥8 unusual mutations, since these 21 sequences were considered to be at high risk of poor sequence quality. We then recalculated the proportion of each amino acid at each position for the remaining 4,242 sequences. The original and aligned sets of sequences for the 4,242 one-per-person filtered sequences from these studies in FASTA format are in gp41NaiveOriginal.fas and gp41NaiveAligned.fas (Data Citation 1). The header for each sequence contains the GenBank accession number and the LANL-designated subtype. The file gp41AAPrevalence.csv (Data Citation 1) lists the proportion of each amino acid at each *gp41* position. In this file insertions, deletions, and mixtures are indicated by “ins”, “del”, and “X”, respectively. Figure 6 displays the distribution of amino acids at each of the 345 *gp41* positions in the one-per-person group M HIV-1 *gp41* sequences from ART-naïve individuals.

### *Gag* and *gp41* selection indexes

We used empirical *gag* and *gp41* amino acid site frequencies to calculate a selection index for each amino acid change that developed during therapy defined as follows: log_10_ of the ratio of the proportion of the pre-therapy amino acid in PI-naïve individuals divided by the proportion of the post-therapy amino acid in PI-naïve individuals (fold change). Amino acid changes with a high selection index were defined as changing from a highly conserved or relatively common amino acid variant at a position to an amino acid with a prevalence at least 10 times less common (i.e., a selection index ≥1.0).

The distribution of all selection indexes for *gag* and *gp41* according to treatment was plotted using an R script that accepts as input the list of amino acid changes between pairs of sequences and data on the proportion of each amino acid in an external database. The script and the resulting figures are located at scripts/make-graphical-summary.r, reports/gag-mutations.pdf, and reports/gp41-mutations.pdf (Data Citation 1). As no statistical model for the expected distribution of selection indexes for proteins under selective drug pressure has been developed, the plots are useful primarily for identifying loci at which changes from a conserved to an unusual amino acid were clustered. As noted in our previous manuscript, we found no discernible difference in either *gag* or *gp41* in the distribution of selection indexes between PI- and NNRTI-treated individuals.

### Code availability

The code used in this manuscript includes the set of Python (version 3.5.2), R (version 3.2.3), and Linux shell scripts that are available on Github (https://github.com/hivdb/gag-gp41) and in the gag-gp41.zip file submitted to Dryad Digital Repository (Data Citation 1). The Github site and the Dryad zip file also includes the 24 files cited in this Data Descriptor. There are no restrictions on accessing or using the code or files as they are released under the open source MIT License.

## Data Records

The original and aligned pre- and post-treatment *gag* and *gp41* sequences from our previous published study are available in four FASTA files in which each sequence header contains four fields: GenBank accession ID, PID, treatment time point, and treatment category (for example KY579846|118827_PIs_Pre). The sequence files, which are named gagOriginal.fas, gagAligned.fas, gp41Original.fas, and gp41Aligned.fas, are located in the directory data/fasta/ (Data Citation 1). Table 1 (available online only) lists the GenBank accession IDs according to PID, treatment time point, and treatment category (Data Citation 2, Data Citations 3, Data Citations 4, Data Citations 5, Data Citations 6, Data Citations 7, Data Citations 8, Data Citations 9, Data Citations 10, Data Citations 11, Data Citations 12, Data Citations 13, Data Citations 14, Data Citations 15, Data Citations 16, Data Citations 17, Data Citations 18, Data Citations 19, Data Citations 20). The file data/insertions.csv (Data Citation 1) lists each of the insertions in *gag* and *gp41* as these were removed during sequence alignment. The Newick representation of the neighbour-joining trees for the aligned *gag* and *gp41* sequences are in the directory data/phylo/ (Data Citation 1). Tables 2, 3, and 4 summarize the differences between pre- and post-treatment sequences for *gag*, the *gag* cleavage sites, and *gp41*, respectively.

The Linux shell scripts that pass parameters to Hyphy batch language scripts used to perform the pairwise dN/dS analysis, FEL diversifying selection analysis, and MEDS directional selection analysis are named run-pairwise.sh, run-fel.sh, and run-meds.sh, respectively (Data Citation 1). They are available in the directory scripts/. The summarized results of the pairwise dN/dS analysis are in Tables 2 and 4. The results of FEL and MEDS are in Tables 5 and 6.

The file data/naiveStudies/gagStudies.csv (Data Citation 1) contains the list of all studies with three or more individuals from whom *gag* sequences were obtained for analyzing mutation prevalence. The files data/naiveStudies/gagNaiveOriginal.fas and data/naiveStudies/gagNaiveAligned.fas (Data Citation 1) contain the 5,338 one-per-person quality-control filtered original and aligned sequences from these studies. Table 7 lists the *gag* signature APOBEC mutations. Figure 1 shows the distribution of the number of *gag* signature APOBEC mutations prior to quality control filtering. Figure 2 shows the distribution of the number of unusual amino acids per *gag* sequence. The file gagAAPrevalence.csv (Data Citation 1) lists the proportion of each amino acid at each *gag* position according to HIV-1 subtype in one-per-person sequences filtered for an excess of signature APOBEC mutations and unusual amino acids. report/gag-naive-indels.pdf (Data Citation 1) displays the distribution of insertions and deletions at each position in *gag*. Figure 3 displays the proportions of all *gag* amino acid variants present at 1.0% or greater frequency of one-per-person sequences.

The file data/naiveStudies/*gp41*Studies.csv (Data Citation 1) contains the list of all studies with five or more individuals from whom *gp41* sequences were obtained for analyzing mutation prevalence. The files data/naiveStudies/gp41NaiveOriginal.fas and data/naiveStudies/gp41NaiveAligned.fas (Data Citation 1) contain the 4,242 one-per-person quality-control filtered original and aligned sequences from these studies. Table 8 lists the *gp41* signature APOBEC mutations. Figure 4 shows how the distribution of the number of *gp41* signature APOBEC mutations prior to quality control filtering. Figure 5 shows the distribution of the number of unusual amino acids per *gp41* sequence. The file gp41AAPrevalence.csv (Data Citation 1) lists the proportion of each amino acid at each *gp41* position according to HIV-1 subtype in one-per-person sequences filtered for an excess of signature APOBEC mutations and unusual amino acids. report/gp41-naive-indels.pdf (Data Citation 1) displays the distribution of insertions and deletions at each position in *gp41*. Figure 6 displays the proportions of all *gp41* amino acid variants present in ≥1.0% of one-per-person sequences.

The file scripts/run-basic.py and scripts/make-graphical-summary.r (Data Citation 1) contain the script that accept a list of amino acid changes between pairs of sequences and data on the proportion of each mutation to generate a plot showing the selection indexes for each mutation. The files reports/gag-mutations.pdf, and reports/gp41-mutations.pdf contain the output of these scripts.

## Technical Validation

Several concerns arise when calculating positional amino acid prevalence from large numbers of sequences in public databases: (i) Do any of the sequences contain nucleotide sequence errors introduced by those who submitted the sequence?; (ii) Do any of the sequences contain annotation errors introduced by those who submitted the sequence or by database curators?; (iii) Have errors been introduced during sequence alignment resulting in the spurious alignment of nonhomologous positions and secondarily inaccurate mutation proportion data?; and (iv) In the case of HIV-1, do the sequences have evidence of APOBEC-mediated G-to-A hypermutation or other evidence for biological artefact consistent with a nonviable virus protein?

In our analyses, we used the LANL HIV Sequence Database^21^ to retrieve complete group M HIV-1 *gag* and *gp41* sequences from previously published studies submitted to GenBank^24^. Despite the fact that GenBank is the standard database for sequences determined by dideoxynucleoside sequencing and that the LANL HIV Sequence Database is a curated HIV sequence database, we performed additional analyses to address the concerns cited in the previous paragraph. This process involved first removing sequences containing large gaps, multiple frame shift mutations, and an excess of signature APOBEC mutations. This was followed by removing a small number of sequences containing high numbers of unusual mutations.

The approach for identifying likely APOBEC-mediated G-to-A hypermutation was similar to an approach that we previously described for HIV-1 protease, RT, and integrase^25^. We first identified 45 *gag* and 47 *gp41* signature APOBEC mutations. Overall, 23 of the signature mutations were stop codons at positions for which the highly conserved consensus amino acid was tryptophan (W) and 69 were highly unusual amino acids at conserved positions that usually occurred in a sequence containing one or more stop codons. The distribution of the number of signature APOBEC mutations per sequence was then used to exclude a small proportion of sequences with ≥3 signature APOBEC mutations: 0.6% for *gag* and 1.7% for *gp41*.

**Figure 7:**
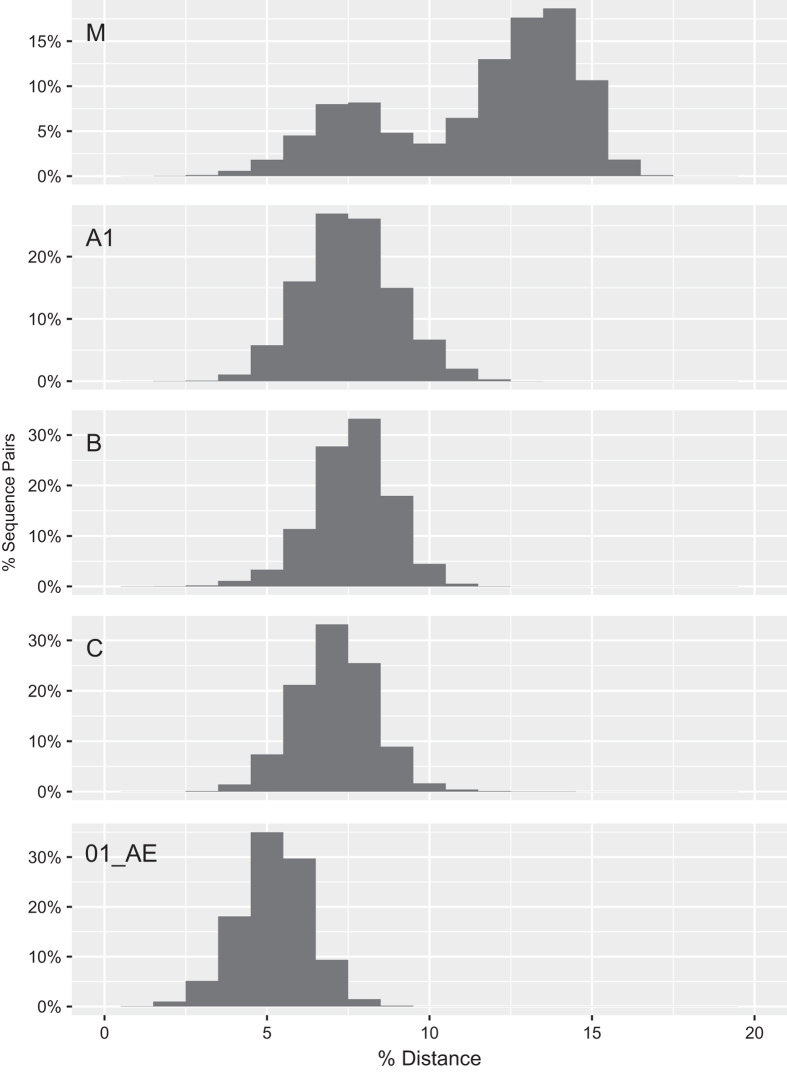
Distribution of pairwise uncorrected nucleotide distances for 5,338 group M HIV-1 *gag* sequences and within each of the four most common subtypes. Approximately 0.001% of group M sequence pairs had distance between 17.5 to 19.6% and cannot be visualized on the figure. The left- and right-sided distributions for the group M sequences reflect intra- and inter-subtype distances, respectively.

**Figure 8:**
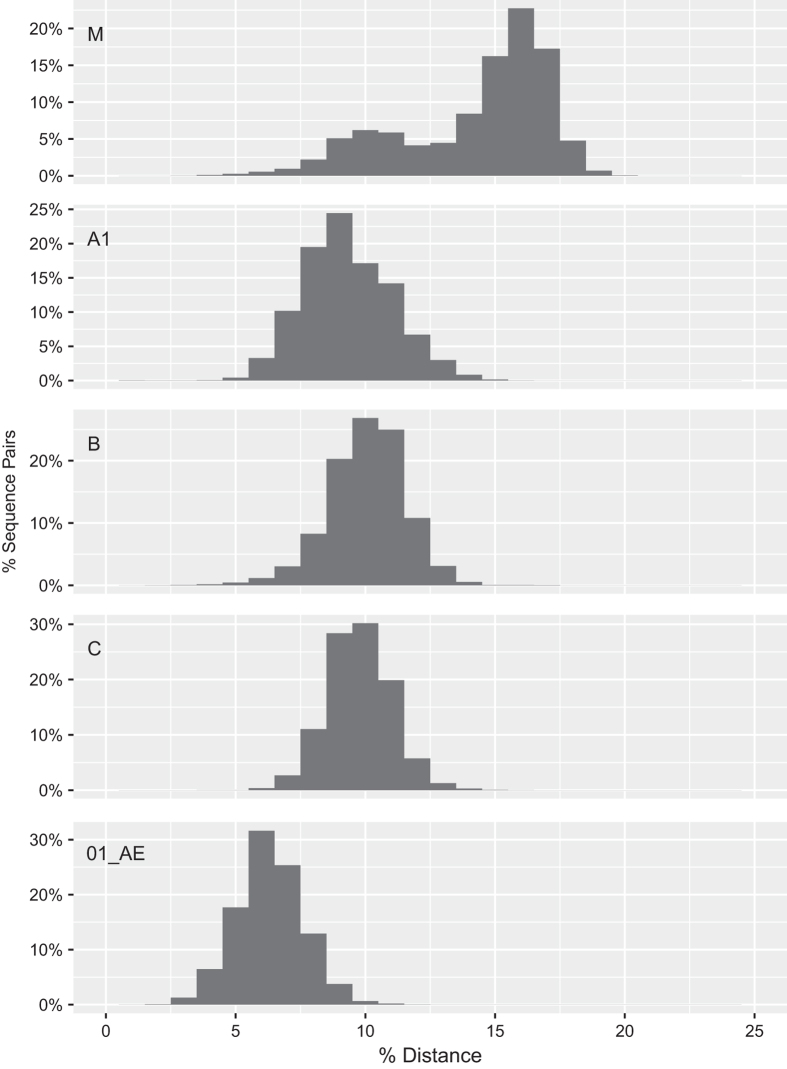
Distribution of pairwise uncorrected nucleotide distances for 4,242 group M HIV-1 *gag* sequences and within each of the four most common subtypes. Approximately 0.001% of group M sequence pairs had distance between 20.5 to 21.3% and cannot be visualized on the figure. The left- and right-sided distributions for the group M sequences reflect intra- and inter-subtype distances, respectively.

Following these steps, we plotted the distribution of pairwise uncorrected nucleotide distances for group M HIV-1 *gag* and *gp41* and for those subtypes for which more than 100 sequences were available (Figs. 7 and 8). The intra- and inter-subtype pairwise distances for both genes clustered around previously reported genetic distances for these genes^26^. The absence of highly divergent *gag* or *gp41* sequences is consistent with our attention to sequence alignment and sequence quality in creating our curated sequence datasets and amino acid profiles.

We also identified a previous study of *gag* amino acid variation and found that 92.9% (914) of the 984 amino acids which we detected at a prevalence of at least 1.0% of PI-naïve individuals were detected among the 993 amino acids detected at a prevalence of at least 1.0% in this earlier study ^27^. This previous study was similar in design to ours with the following exceptions: (i) Sequences were obtained from all published studies through 2012 regardless of whether the individuals from whom the sequences were obtained were PI-experienced, PI-naïve, or of uncertain PI treatment history; (ii) Hypermutated sequences were excluded using the Los Alamos Hypermut tool^28^; and (iii) No isolates were excluded solely on the basis of having a large number of unusual mutations. We did not identify a similarly large study of *gp41* amino acid variation.

The exclusion of outlier sequences is a logical approach to creating useful sequence sets and alignments from which mutation proportion data can be calculated. Nonetheless, highly unusual sequences may not always reflect erroneous or artefactual data. As part of our technical validation pipeline, we have identified those sequences that were excluded should other researchers be interested in their analysis.

## Usage Notes

The complete set of files including tables, figures, sequence files, tab-delimited files, and code files are available as a Dryad data citation and on the GitHub repository. The Dryad data citation provides a stable permanent snapshot of the analyses described in this manuscript. The GitHub repository will evolve as new studies are published and as more published studies are reviewed to expand the sets of filtered annotated *gag* and *gp41* sequences from PI-naïve individuals.

Other researchers sequencing *gag* and/or *gp41* sequences before and after PI therapy will be able to pool our data with theirs and to perform the same dN/dS and selection index analyses using the software described in this manuscript and provided on Dryad and GitHub.

Several other aspects of our data and code will be useful to other researchers even if they are not planning to perform the same analyses described in this manuscript: (i) the signature APOBEC mutations for *gag* and *gp41* will be useful for the study of sequences of these two genes; (ii) the *gag* and *gp41* sequence sets, publication summaries, and mutation prevalence files will also be useful to other researchers studying these genes; and (iii) the Hyphy and shell scripts will be useful to other researchers performing dN/dS analyses on sequence pairs. In particular, the HyPhy script for pairwise analysis has not been previously published.

## Additional information

**How to cite this article**: Tzou, P. L. *et al.* Selection analyses of paired HIV-1 gag and gp41 sequences obtained before and after antiretroviral therapy. *Sci. Data* 5:180147 doi: 10.1084/sdata.2018.147 (2018).

**Publisher’s note**: Springer Nature remains neutral with regard to jurisdictional claims in published maps and institutional affiliations.

## Supplementary Material



## Figures and Tables

**Table 1:** GenBank accessions for each of the paired protease, *gag* and *gp41* sequences

**Group**	**Subject**	**Protease (PR)**		**Gag**		**Gp41**	
		**Baseline**	**Follow-up**	**Baseline**	**Follow-up**	**Baseline**	**Follow-up**
PI	8006	KT340026	KT340027	NA	NA	KT339970	KT339971
PI	14728	KT340028	KT340029	NA	NA	KT339972	KT339973
PI	14736	GQ210720	GQ213759	NA	NA	KT339974	KT339975
PI	18380	AY798294	GQ213798	NA	NA	KT339976	KT339977
PI	24950	KT340030	KT340031	NA	NA	KT339978	KT339979
PI	25082	GQ210971	GQ212432	NA	NA	KT339980	KT339981
PI	26307	KT340032	KT340033	KT339954	KT339955	KT339982	KT339983
PI	38099	KT340034	KT340035	NA	NA	KT339985	KT339984
PI	39143	KT340036	KT340037	KT339958	KT339959	KT339986	KT339987
PI	39270	MG171048	MG171049	NA	NA	KY579942	KY579943
PI	42080	KT340039	KT340040	KT339960	KT339961	KT339988	KT339989
PI	42654	KT340041	KT340042	NA	NA	KT339990	KT339991
PI	56120	KT340047	KT340048	KT339966	KT339967	KT339994	KT339995
PI	56141	KT340049	KT340050	NA	NA	KT339996	KT339997
PI	57479	KT340051	KT340052	NA	NA	KT339998	KT339999
PI	118724	MG171059	MG171060	NA	NA	NA	NA
PI	118745	MG171063	MG171064	KY579860	KY579861	KY579932	KY579933
PI	118747	MG171065	MG171066	NA	NA	NA	NA
PI	118754	MG171067	MG171068	NA	NA	NA	NA
PI	118761	MG171069	MG171070	KY579878	KY579879	NA	NA
PI	118770	MG171071	MG171072	NA	NA	NA	NA
PI	118792	MG171073	MG171074	KY579870	KY579871	NA	NA
PI	118811	MG171075	MG171076	KY579872	KY579873	NA	NA
PI	118820	MG171077	MG171078	NA	NA	NA	NA
PI	118823	MG171079	MG171080	KY579864	KY579865	NA	NA
PI	118827	MG171081	MG171082	KY579846	KY579847	NA	NA
PI	118828	MG171083	MG171084	KY579854	KY579855	NA	NA
PI	118840	MG171085	MG171086	NA	NA	KY579940	KY579941
PI	118846	MG171087	MG171088	KY579880	KY579881	KY579938	KY579939
PI	118849	MG171089	MG171090	KY579884	KY579885	NA	NA
PI	118853	MG171091	MG171092	KY579866	KY579867	NA	NA
PI	118855	MG171093	MG171094	NA	NA	NA	NA
PI	118856	MG171095	MG171096	NA	NA	KY579944	KY579945
PI	118860	MG171097	MG171098	KY579848	KY579849	KY579920	KY579921
PI	118865	MG171099	MG171100	NA	NA	NA	NA
PI	118870	MG171101	MG171102	NA	NA	NA	NA
PI	118886	MG171103	MG171104	KY579868	KY579869	NA	NA
PI	118899	MG171105	MG171106	NA	NA	KY579924	KY579925
PI	118903	MG171107	MG171108	KY579874	KY579875	KY579936	KY579937
PI	118905	MG171109	MG171110	NA	NA	NA	NA
PI	118910	MG171111	MG171112	NA	NA	KY579922	KY579923
PI	118918	MG171113	MG171114	NA	NA	NA	NA
PI	118925	MG171115	MG171116	KY579876	KY579877	NA	NA
PI	118930	MG171117	MG171118	NA	NA	NA	NA
PI	118934	MG171119	MG171120	NA	NA	NA	NA
PI	118935	MG171121	MG171122	KY579850	KY579851	KY579928	KY579929
PI	118942	MG171123	MG171124	NA	NA	NA	NA
PI	118951	MG171125	MG171126	NA	NA	KY579926	KY579927
PI	118954	MG171127	MG171128	KY579858	KY579859	NA	NA
PI	118956	MG171129	MG171130	KY579882	KY579883	NA	NA
PI	118962	MG171131	MG171132	NA	NA	NA	NA
PI	118965	MG171133	MG171134	NA	NA	KY579946	KY579947
PI	118972	MG171135	MG171136	KY579862	KY579863	KY579934	KY579935
PI	118973	MG171137	MG171138	KY579852	KY579853	KY579930	KY579931
PI	118982	MG171139	MG171140	NA	NA	NA	NA
PI	118985	MG171141	MG171142	NA	NA	NA	NA
PI	118986	MG171143	MG171144	KY579856	KY579857	NA	NA
NNRTI	8349	GQ206503	MG171044	NA	NA	KY579904	KY579905
NNRTI	9918	GQ206632	MG171045	KY579838	KY579839	KY579906	KY579907
NNRTI	25036	GQ210904	MG171046	KY579840	KY579841	KY579908	KY579909
NNRTI	35596	GQ212974	KY190132	KY579842	KY579843	KY579910	KY579911
NNRTI	37879	MG171047	KY190134	KY579844	KY579845	KY579912	KY579913
NNRTI	42036	MG171050	KY190141	KY579814	KY579815	KY579886	KY579887
NNRTI	42183	MG171051	MG171052	KY579816	KY579817	KY579888	KY579889
NNRTI	55928	MG171053	MG171054	KY579818	KY579819	KY579890	KY579891
NNRTI	57448	MG171055	KY190153	NA	NA	KY579914	KY579915
NNRTI	61483	MG171056	MG171057	NA	NA	KY579916	KY579917
NNRTI	61631	MG171058	KY190163	NA	NA	KY579918	KY579919
NNRTI	252392	MG171061	MG171062	KY579832	KY579833	KY579898	KY579899
NNRTI	264159	KY787124	KY787125	KY579836	KY579837	KY579902	KY579903
NNRTI	108149	KY787112	KY787113	KY579824	KY579825	NA	NA
NNRTI	232768	KY787118	KY787119	KY579830	KY579831	NA	NA
NNRTI	122034	KY787114	KY787115	KY579826	KY579827	KY579894	KY579895
NNRTI	214046	KY787116	KY787117	KY579828	KY579829	KY579896	KY579897
NNRTI	21890	KY787108	KY787109	KY579820	KY579821	KY579892	KY579893
NNRTI	253540	KY787122	KY787123	KY579834	KY579835	KY579900	KY579901
NNRTI	44969	KY787110	KY787111	KY579822	KY579823	NA	NA
Abbreviations: PI: Individuals who received a ritonavir-boosted protease inhibitor containing regimen. NNRTI: Individuals who received a nonnucleoside RT inhibitor (NNRTI) containing regimen. NA: not applicable as a sequence was not performed							

**Table 2:** Pairwise nucleotide and amino acid changes, dN/dS ratios, and percent ambiguities in HIV-1 *gag* before and after protease inhibitor (PI) and nonnucleoside RT inhibitor (NNRTI) therapy.

	**PI (n=24)**	**NNRTI (n=16)**	**P**^a^
***Median % pairwise NA changes (interquartile range)***			
Complete gene	1.1 (0.8–1.6)	0.6 (0.5–1.3)	0.1
Matrix	0.4 (0.2–0.6)	0.2 (0.1–0.6)	0.1
C-terminal region	0.3 (0.2–0.6)	0.3 (0.2–0.3)	0.6
***Median % pairwise AA changes (interquartile range)***			
Complete gene	1.4 (0.8–1.8)	0.8 (0.6–1.2)	0.1
Matrix	0.6 (0.4–0.6)	0.4 (0.4–0.9)	1.0
C-terminal region	0.7 (0.2–0.9)	0.4 (0.4–0.8)	0.6
***Median pairwise dN/dS ratio (interquartile range)***^b^			
Complete gene	0.21 (0.08–0.48)	0.35 (0.15–0.57)	0.2
Matrix	0.24 (0.00–0.68)	0.79 (0.16–∞)	0.1
C-terminal region	0.46 (0.19–1.14)	0.50 (0.24–1.05)	0.9
***Median % IUPAC Ambiguities (interquartile range)***^c^			
Baseline (complete gene)	0.4^c^ (0.1–0.8)	0.1 (0.1–0.1)	0.1
Follow-up (complete gene)	0.0^c^ (0.0–0.6)	0.0 (0.0–0.1)	0.2

^a^Mann-Whitney U Test.

^b^Ratio of nonsynonymous to synonymous mutations.

^c^The proportion of ambiguities (i.e., mixtures of more than one base at the same position) was significantly higher at baseline than at follow-up in the PI group (p=0.02; Mann-Whitney U Test).

**Table 3:** Pairwise nucleotide and amino acid changes, dN/dS ratios, and percent ambiguities in HIV-1 *gp41* before and after protease inhibitor (PI) and nonnucleoside inhibitor (NNRTI) therapy.

	**PI (n=28)**	**NNRTI (n=17)**	**P**^a^
***Median % pairwise NA changes (interquartile range)***			
Complete gene	1.1 (0.5–1.5)	0.8 (0.6–1.4)	0.8
Cytoplasmic domain	0.4 (0.3–0.9)	0.6 (0.2–0.7)	1.0
***Median % pairwise AA changes (interquartile range)***			
Complete gene	1.4 (0.6–2.2)	1.3 (0.9–2.2)	0.7
Cytoplasmic domain	0.9 (0.3–1.4)	0.9 (0.6–1.1)	0.9
***Median pairwise dN/dS ratio (interquartile range)***^b^			
Complete gene	0.42 (0.16–0.74)	0.44 (0.29–0.96)	0.4
Cytoplasmic domain	0.59 (0.17–1.99)	0.47 (0.32–0.93)	0.9
***Median % IUPAC Ambiguities (interquartile range)***			
Baseline (complete gene)	0.1 (0.0–0.2)	0.1 (0.0–0.1)	0.8
Follow-up (complete gene)	0.0 (0.0–0.1)	0.0 (0.0–0.1)	0.4

^a^Mann-Whitney U Test.

^b^Ratio of nonsynonymous to synonymous mutations.

**Table 4:** Amino acid changes occurring within protease cleavage sites in *gag* during protease inhibitor (PI) and nonnucleoside RT inhibitor (NNRTI) therapy.

**Cleavage site**	**Position**	**Baseline AAs**	**Follow-up AAs**	**# patients**
***PI group***				
SP1 / Nucleocapsid (NC)	373	IPAAM|MQRGN	MPAAM|MQRGN	1
	373	PTAIM|MQKGN	STAIM|MQKGN	1
	373, 375	STAIM|MQKGN	PTTIM|MQKGN	1
	374, 380	PXAIM|MQKGN^a^	PPAIM|MQRGN	1
	374, 381	SAAMM|MQRSN	STAMM|MQRGN	1
	374	SXAIM|MQKGN^a^	STAIM|MQKGN	1
	375, 378	SANIM|MQRGN	SAAIM|IQRGN	1
	376, 380	SATIM|MQKGN	SATTM|MQRGN	1
	378	SASVM|MQRGN	SASVM|IQRGN	1
Nucleocapsid (NC) / SP2	429, 436	EKQAN|FLGRI	ERQAN|FLGKI	1
	436	ERQAN|FLGKL	ERQAN|FLGRL	1
	436	ERQAN|FLGXI^a^	ERQAN|FLGKI	1
	437	ERQAN|FLGKX^a^	ERQAN|FLGKL	1
SP2 / p6^*g*^^a^^*g*^	453	RPGNF|LQSRL	RPGNF|LQSRP	2
p6^*pol*^ / Protease^b^	485, 486, 487, 490	VXLXF|PXITL^a^	VSFSF|PQITL	1
	486	VSVNF|PQITL	VSLNF|PQITL	1
***NNRTI group***				
Matrix (MA) / Capsid (CA)	132	VSHNY|PIVQN	VSHNF|PIVQN	1
SP1 / Nucleocapsid (NC)	374	-STAM|MQRGN	-TTAM|MQRGN	1
	374	PTTIM|MQRGN	PATIM|MQRGN	1
	375	PAAIM|MQRGN	PATIM|MQRGN	1
	375	SAAIM|MQKGN	SANIM|MQKGN	1
	375	STAIM|MQRGN	STTIM|MQRGN	1
Nucleocapsid (NC) / SP2	429	EKQAN|FLGRL	ERQAN|FLGRL	1
SP2 / p6^*g*^^a^^*g*^	453	RPGNF|PQSRL	RPGNF|PQSRP	1
p6^*pol*^ / Protease^b^	487	VSFSF|PQITL	VSFNF|PQITL	1
	488	VSLDL|PQITL	VSLDF|PQITL	1

^a^X stands for mixtures consisted of at least two amino acids which were not subtype B consensus.

^b^A -1 frameshift was applied to the *pol* reading frame relative to the *gag* reading frame.

**Table 5:** Amino acid positions with evidence of diversifying selection and mutations with evidence of directional selection in HIV-1 *gag* within the protease inhibitor (PI) and nonnucleoside RT inhibitor (NNRTI)-treatment groups.

***Positions with Evidence of Diversifying Selection (FEL)***^a^	
PI	67 (0.04), 115 (0.05), 223 (0.008), 468 (0.03), 469 (0.008), 474 (0.03)
NNRTI	54 (0.04), 69 (0.03), 173 (0.04)
***Mutations with Evidence of Directional Selection (MEDS)***^b^	
PI	K59M (1, p=0.000), Q219H (3, p=0.000)^c^, F370Y (1, p=0.000), T371N (1, p=0.001)
NNRTI	Y79F (2, p=0.000), K110M (1, p=0.001), A371T (1, p=0.001), N371T (1, p=0.001)

^a^Parentheses contain p-values.

^b^Parentheses contain number of individuals and p-values.

^c^In one individual, there was a change from H219→Q.

**Table 6:** Amino acid positions with evidence of diversifying selection and the mutations with evidence of directional selection in HIV-1 *gp41* within the protease inhibitor (PI) and nonnucleoside RT inhibitor (NNRTI)-treatment groups.

***Positions with Evidence of Diversifying Selection (FEL)***^a^	
PI	55 (0.02), 101 (0.01), 273 (0.02)
NNRTI	24 (0.009), 187 (0.04), 310 (0.04)
***Mutations with Evidence of Directional Selection (MEDS)***^b^	
PI	T307I (3, p=0.001), I325F (1, p=0.001), L325F (1, p=0.001)
NNRTI	None

^a^Parentheses contain p-values.

^b^Parentheses contain number of individuals and p-values.

**Table 7:** HIV-1 *gag* signature APOBEC mutations^a^.

**Position**	**Consensus AA**^b^	**Consensus %**^b^	**Signature mutation**^c^	**Proportion occurring in sequence with a stop codon**	**# Sequences with mutation**
1	M	98.9	I	89	36
16	W	98.7	*	100	31
24	G	99.2	E	53	17
25	G	99.2	R	71	7
36	W	99.6	*	100	15
56	G	99.9	R	60	5
71	G	99.5	R	75	4
99	E	99.3	K	67	6
140	G	99.8	E	100	1
140	G	99.8	R	82	11
155	W	99.5	*	100	31
192	G	99.8	E	60	5
192	G	99.8	R	90	10
212	W	99.4	*	100	33
214	R	99.8	K	58	12
221	G	99.9	R	78	9
229	R	99.6	K	67	18
232	R	99.1	K	60	25
233	G	99.8	R	100	5
249	W	99.5	*	100	22
265	W	99.6	*	100	25
269	G	99.7	R	80	15
284	D	99.5	N	100	3
288	G	99.6	R	88	24
294	R	99.7	K	67	15
298	D	99.8	N	67	3
299	R	99.8	Q	100	5
305	R	99.8	K	67	6
316	W	99.5	*	100	31
338	G	99.7	R	53	19
352	G	99.8	R	71	7
354	G	99.8	R	70	10
355	G	99.9	R	60	5
365	E	99.8	K	100	2
396	G	100	S	100	2
399	G	99.7	R	71	17
414	W	99.3	*	100	31
417	G	99.4	R	71	24
420	G	99.7	E	67	6
420	G	99.7	R	62	8
435	G	99.3	R	72	29
438	W	99.5	*	100	4
443	G	98.8	R	60	10
446	G	99.4	E	60	10
446	G	99.4	R	76	21

^a^Mutations at highly conserved positions that are likely to occur in sequences with stop codons and that occur in an appropriate dinucleotide context: GG→AG or GA→AA.

^b^Amino acid present in >97.5% of Group M HIV-1 sequences.

^c^Mutations strongly consistent with APOBEC-mediated G-to-A hypermutation. “*”: stop codon.

**Table 8:** HIV-1 *gp41* signature APOBEC mutations^a^.

**Position**	**Consensus AA**^b^	**Consensus %**^b^	**Signature mutation**^c^	**Proportion occurring in sequence with a stop codon**	**# Sequences with mutation**
5	G	98.4	R	87	39
10	G	98.3	R	90	69
16	G	99.2	E	62	13
16	G	99.2	R	83	35
19	M	98.6	I	93	95
36	G	99	R	100	5
36	G	99	S	93	44
60	W	98.5	*	100	103
61	G	99.4	S	83	6
68	R	99.5	K	92	25
73	E	99.4	K	69	32
78	D	99.3	N	83	18
83	G	98.5	E	71	7
83	G	98.5	R	86	58
85	W	98.4	*	100	100
86	G	99.7	R	100	1
86	G	99.7	S	93	15
89	G	98.1	E	65	20
89	G	98.1	R	91	70
99	W	98.4	*	100	102
103	W	98.6	*	100	93
112	W	98.3	*	100	107
117	W	98.5	*	100	99
120	W	98.2	*	100	117
146	E	99.2	K	55	40
155	W	98.7	*	100	85
159	W	98.3	*	100	99
161	W	98.6	*	100	87
167	W	99.3	*	100	41
169	W	98.2	*	100	66
179	G	97.6	E	57	7
179	G	97.6	R	96	54
180	G	98.2	R	100	2
183	G	98.4	R	69	16
183	G	98.4	S	83	60
200	G	98.7	E	60	15
200	G	98.7	R	90	69
227	G	98.8	E	79	19
227	G	98.8	R	90	48
240	G	98.1	E	75	12
240	G	98.1	R	82	72
246	W	98.2	*	100	110
248	D	99.7	N	82	11
269	R	98.3	K	57	53
292	W	97.8	*	100	108
339	G	99.1	S	65	34
341	E	99	K	55	53

^a^Mutations at highly conserved positions that are likely to occur in sequences with stop codons and that occur in an appropriate dinucleotide context: GG→AG or GA→AA.

^b^Amino acids present in >97.5% of Group M HIV-1 sequences.

^c^Mutations strongly consistent with APOBEC-mediated G-to-A hypermutation. “*”: stop codon.
